# A Homolog of FHM2 Is Involved in Modulation of Excitatory Neurotransmission by Serotonin in *C. elegans*

**DOI:** 10.1371/journal.pone.0010368

**Published:** 2010-04-28

**Authors:** Elena G. Govorunova, Mustapha Moussaif, Andrey Kullyev, Ken C. Q. Nguyen, Thomas V. McDonald, David H. Hall, Ji Y. Sze

**Affiliations:** 1 Department of Molecular Pharmacology, Albert Einstein College of Medicine, Bronx, New York, United States of America; 2 Department of Medicine, Albert Einstein College of Medicine, Bronx, New York, United States of America; 3 Department of Neuroscience, Albert Einstein College of Medicine, Bronx, New York, United States of America; Columbia University, United States of America

## Abstract

The *C. elegans eat-6* gene encodes a Na^+^, K^+^-ATPase α subunit and is a homolog of the familial hemiplegic migraine candidate gene FHM2. Migraine is the most common neurological disorder linked to serotonergic dysfunction. We sought to study the pathophysiological mechanisms of migraine and their relation to serotonin (5-HT) signaling using *C. elegans* as a genetic model. In *C. elegans*, exogenous 5-HT inhibits paralysis induced by the acetylcholinesterase inhibitor aldicarb. We found that the *eat-6(ad467)* mutation or RNAi of *eat-6* increases aldicarb sensitivity and causes complete resistance to 5-HT treatment, indicating that EAT-6 is a component of the pathway that couples 5-HT signaling and ACh neurotransmission. While a postsynaptic role of EAT-6 at the bodywall NMJs has been well established, we found that EAT-6 may in addition regulate presynaptic ACh neurotransmission. We show that *eat-6* is expressed in ventral cord ACh motor neurons, and that cell-specific RNAi of *eat-6* in the ACh neurons leads to hypersensitivity to aldicarb. Electron microscopy showed an increased number of synaptic vesicles in the ACh neurons in the *eat-6(ad467)* mutant. Genetic analyses suggest that EAT-6 interacts with EGL-30 Gαq, EGL-8 phospholipase C and SLO-1 BK channel signaling to modulate ACh neurotransmission and that either reduced or excessive EAT-6 function may lead to increased ACh neurotransmission. Study of the interaction between *eat-6* and 5-HT receptors revealed both stimulatory and inhibitory 5-HT inputs to the NMJs. We show that the inhibitory and stimulatory 5-HT signals arise from distinct 5-HT neurons. The role of *eat-6* in modulation of excitatory neurotransmission by 5-HT may provide a genetic explanation for the therapeutic effects of the drugs targeting 5-HT receptors in the treatment of migraine patients.

## Introduction

Serotonin (5-hydroxytryptamine, 5-HT) functions as a neuromodulator by inhibiting and enhancing synaptic transmission of other neurotransmitters. Dysregulation of 5-HT signaling has been implicated in pathophysiology of many disorders, including migraine [1]. Migraine is a polygenic trait. Familial hemiplegic migraine (FHM) is a rare autosomal dominant subtype of migraine associated with genetic lesions in three genes, FHM1 encoding the α1 subunit of the voltage-gated Ca^2+^ channel CACNA1A, FHM2 encoding the α2 subunit of the Na^+^,K^+^-ATPase ATP1A2, and FHM3 encoding the α1 subunit of the voltage-gated Na^+^ channel SCN1A [2]. According to the current view, mutations in these genes result in excessive excitatory neurotransmission in the brain leading to migraine pain [3]. However, verification of this hypothesis, as well as analysis of the relationship between migraine and 5-HT signaling is difficult due to the complexity of mammalian nervous systems. The nematode *C. elegans* is an attractive genetic model to study neuronal functions *in vivo*. The first two FHM genes are conserved in this organism and are designated as *unc-2* and *eat-6*, respectively. Analysis of *unc-2* mutants has been undertaken for modeling of FHM1 migraine channelopathy [4]. In this study, we focused on the FHM2 homolog *eat-6* to gain new insights into biochemical and physiological mechanisms of migraine pathology.

The Na^+^,K^+^-ATPase is a sodium pump that uses energy derived from ATP hydrolysis to extrude cytoplasmic Na^+^ in exchange for extracellular K^+^ across the plasma membrane [5]. In human there are four genes encoding Na^+^,K^+^-ATPase α subunits [6]. The α1 isoform is the ubiquitous housekeeping enzyme. FHM2 is the α2 isoform, which together with the α3 isoform is enriched in neuronal and muscular systems, and the α4 isoform is confined to testis. In the adult brain α2 is mostly expressed in glia; besides, it is present in skeletal muscle, heart and other tissues. The *C. elegans* genome harbors four other Na^+^,K^+^-ATPase α subunit genes besides *eat-6* [7]. EAT-6 appears to be the closest functional homolog of FHM2, as no other α subunit genes are expressed in excitable cells [8]. EAT-6 and FHM2 share 72% identity, 84% homology at the protein level. A homozygous *eat-6* deletion mutant is reported to be embryonic lethal/early larval arrest (http://www.wormbase.org). Four other *eat-6* mutations were initially isolated for feeble contractions and delayed relaxations of the pharyngeal muscles [9], and subsequently shown to affect the membrane potential of these cells [10]. A recent study demonstrated that *eat-6* is localized at the NMJs in the bodywall muscles and regulates synaptic efficacy [8]. Taken together, these studies convinced us that further research into *eat-6* function in *C. elegans* might provide a deeper insight into the mechanisms of migraine in humans.

Here we provide evidence that *eat-6* may also act in locomotory cholinergic neurons, and that mutations in *eat-6* disrupt inhibitory 5-HT input to the control of excitatory synaptic transmission at *C. elegans* bodywall NMJs.

## Results

### Molecular lesions of *eat-6* alleles

**Figure 1:**
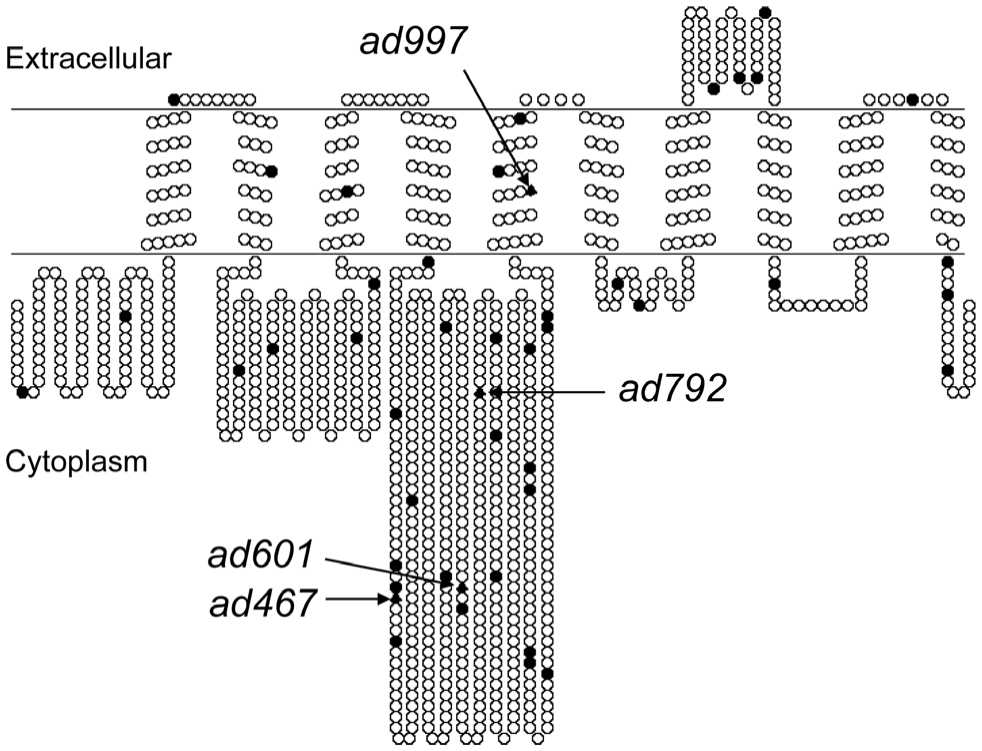
Predicted topology of EAT-6/FHM2 and location of mutations. A schematic depiction of the human FHM2 protein generated by the TOPO2 program (http://www.sacs.ucsf.edu/TOPO2). The sites of mutations found in migraine patients [13] are shown by black circles. Corresponding residues of *eat-6* mutations are shown by black triangles.

Sequencing of mutant genomic DNA confirmed that *ad467*, *ad601* and *ad997* alleles are missense mutations leading to the following amino acid substitutions in the EAT-6 protein: L359F, G522E and S761F, respectively [11]. In addition, we identified that the *ad792* allele carries a missense mutation that results in the R569H substitution. All four *eat-6* mutations substitute amino acid residues that are conserved in human FHM2. Three out of four mutated residues are in the intracellular 4–5 loop (Figure 1), which contains the nucleotide-binding domain and the phosphorylation site essential for the enzymatic activity [5], [12]. The majority of mutations found in human migraine patients are also located in this region [13]. The sites of the *ad467* and *ad601* mutations are very close to the sites of pathological human mutations, and the *ad792* mutation substitutes the same residue as the FHM2(R593W) mutation [14].

### *eat-6(ad467)* mutant is hypersensitive to aldicarb and does not respond to 5-HT

**Figure 2:**
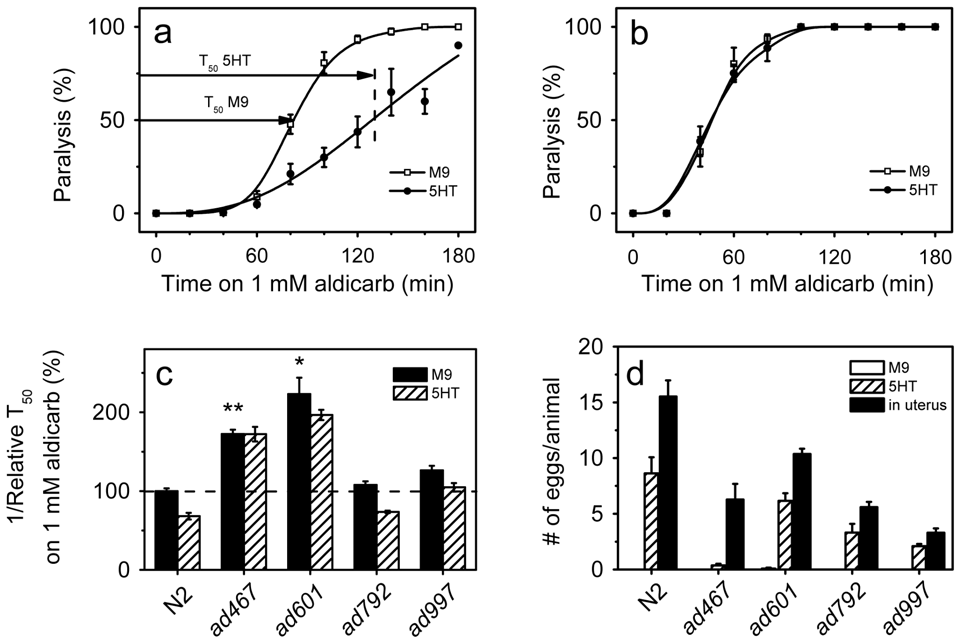
*eat-6(ad467)* is hypersensitive to aldicarb and resistant to 5-HT. (**a**) and (**b**) The time course of aldicarb-induced paralysis of animals pretreated with 5 mg/ml 5-HT (black circles) and without 5-HT treatment (open squares) in wild type (**a**) and *eat-6(ad467)* animals (**b**). The error bars indicate SEM (n>3 replicates). T_50_ is the time course when 50% of the worms were paralyzed. We use reciprocal T_50_ values as a measurement for the rate of paralysis. (**c**) Relative reciprocal T_50_ values of aldicarb-induced paralysis of worms pretreated with 5-HT (hatched bars) and those without 5-HT treatment (black bars). The values of 5-HT-treated WT worms and mutants are normalized to that of WT worms without 5-HT treatment. The error bars indicate SEM (n>3 replicates). *ad467* and *ad601* were hypersensitive to aldicarb compared to WT (* p<0.05, ** p<0.001). 5-HT treatment significantly reduced the rate of aldicarb-induced paralysis in WT and *ad792* animals, compared to their untreated siblings (p<0.0005), whereas no significant reduction by 5-HT treatment was found in the *ad467*, *ad601* and *ad997* alleles. Student's *t*-test. (**d**) Influence of 5-HT on egg laying in *eat-6* mutant alleles. The bars show the number of eggs in the uterus (black bars), and the number of eggs laid per h per animal incubated in M9 buffer (open bars) and in M9 buffer supplemented with 5 mg/ml 5-HT (hatched bars). *C. elegans* generally does not lay eggs in M9 [18]. The error bars indicate SEM (n>3 replicates, 8 animals of each genotype for each condition per trial).

Aldicarb, an inhibitor of acetylcholinesterase, causes paralysis in *C. elegans* due to accumulation of acetylcholine (ACh) at the locomotory NMJs, and is therefore frequently used to measure the steady state ACh release in living *C. elegans* animals [15], [16]. In WT animals exogenous 5-HT slowed down the paralytic effect of aldicarb (Figure 2a). By contrast, 5-HT treatments did not reduce the sensitivity to paralysis induced by levamisole, a specific agonist of the nicotinic ACh receptor UNC-29 in the bodywall muscles (data not shown) [17]. These results suggest that 5-HT signaling inhibits ACh release by the motor neurons [17]. Out of four tested *eat-6* alleles, the *ad467* mutant showed the strongest defects in the function of the pharyngeal muscles [10]; we found that *ad467* animals were hypersensitive to aldicarb and completely resistant to 5-HT treatment (Figure 2b,c). The *ad997* and *ad792* mutations that modestly disrupted the function of the pharyngeal muscles [10] did not cause significant hypersensitivity to aldicarb, although the *ad997* mutation reduced the response to 5-HT treatment (Figure 2c). Interestingly, the *ad601* mutation that least affected the function of the pharyngeal muscles [10] caused the strongest hypersensitivity to aldicarb (Figure 2c) [8].

To further characterize the effect of *eat-6* mutations on sensitivity to 5-HT, we tested the egg-laying response of the mutants to 5-HT treatment. In WT animals, exogenous 5-HT stimulated egg laying (Figure 2d) [18]. The *ad467* mutant laid practically no eggs in response to 5-HT, whereas all the other three *eat-6* alleles laid some (Figure 2d, hatched bars). To exclude the possibility that the failure of *ad467* animals to lay eggs in response to 5-HT was simply due to a reduced number of fertilized eggs, we counted the number of eggs in the uterus of one-day old adult hermaphrodite worms. Although all four *eat-6* alleles had reduced number of eggs in the uterus, *ad467* worms carried at least as many fertilized eggs as *ad792* and *ad997* mutants (Figure 2d, black bars). Therefore, we concluded that the *eat-6(ad467)* mutation disrupts 5-HT regulation in the egg-laying circuit as well as 5-HT-induced modulation of ACh neurotransmission at the bodywall NMJs. The differential effects of the *eat-6* mutations on the function of the pharyngeal muscles [10], the sensitivity to aldicarb and the responses to 5-HT suggest allele-specific modification of EAT-6 function in particular cellular mechanisms, rather than a simple reduction of its activity.

### Both reduction and excess of EAT-6 protein cause hypersensitivity to aldicarb and resistance to 5-HT

**Figure 3:**
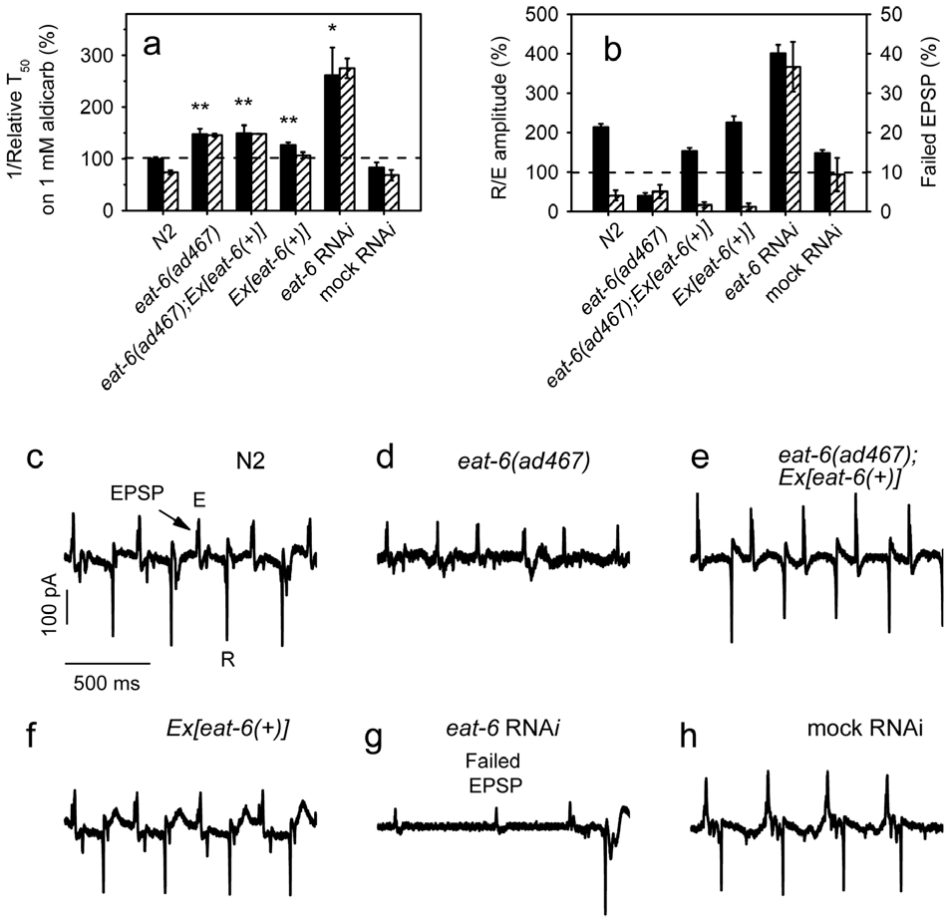
Phenotypic analysis of *eat-6* RNAi and overexpression. (**a**) Relative reciprocal T_50_ values of aldicarb-induced paralysis of animals untreated (black bars) or pretreated with 5-HT (hatched bars). The error bars indicate SEM (n>3 replicates). Both RNAi and overexpression of *eat-6* conferred hypersensitivity to aldicarb compared to WT (* p<0.05, ** p<0.01). Pretreatment of 5-HT reduced aldicarb sensitivity in WT animals (p<0.005), but not in *eat-6* RNAi animals. Student's t-test. (**b**) The ratio of the amplitude of the excitation and recovery peaks (black bars, left axis), and the fraction of EPSPs that failed to trigger APs (hatched bars, right axis). The error bars indicate SEM (n = 15). (**c–h**) Representative EPG recordings. EPSP designates an excitatory postsynaptic potential, E designates the peak corresponding to the muscle excitation (depolarization), R designates the peak corresponding to muscle recovery.

To further investigate the relationship between EAT-6 function and phenotypes seen in *eat-6* mutants, we used RNA interference (RNAi) to knock down *eat-6* gene activity. The strain *rrf-3(pk1426)* has enhanced neuronal sensitivity to RNAi [19]. The *rrf-3* worms fed with *E. coli* expressing dsRNA targeted to *eat-6*, but not the empty vector (mock RNAi), showed abnormalities similar to, but stronger than those seen in the *eat-6* mutants. They had a smaller body size, a reduced number of eggs in the uterus, hypersensitivity to aldicarb and resistance to 5-HT (data not shown; Figure 3a), indicating that a reduction of EAT-6 function brings about all these phenotypes.

We next sought to rescue *eat-6* mutant phenotypes by a WT *eat-6* transgene, *Ex[eat-6(+)]*. As it has been shown previously [10], this transgene rescued the pharyngeal defects of the *ad467* mutant (Figure 3b,e). However, we found that the same transgenic animals remained hypersensitive to aldicarb and resistant to 5-HT (Figure 3a). One possible reason for this could be less efficient expression of the *eat-6* transgene in the locomotory system. But, when we crossed this transgene into WT background, we observed an increased sensitivity to aldicarb in the transgenic animals compared to WT animals (Figure 3a). This result suggests that overexpression of *eat-6* may also perturb ACh synaptic transmission at the locomotory NMJs.

Extrachromosomal arrays usually carry multiple copies of the transgene, so to test whether the aldicarb hypersensitivity of the *eat-6* mutant can be corrected by a single copy of the WT gene, we took advantage of the *nT1* genetic balancer, in which part of the chromosome V carrying the *eat-6* locus is translocated to the chromosome IV [20]. The homozygous *eat(ad467)* mutant carrying a single copy of the *nT1* balancer contains two copies of the *eat-6(ad467)* mutant gene and one copy of the WT *eat-6* gene from the balancer; these animals showed significantly reduced aldicarb hypersensitivity, compared to the *eat(ad467)* mutant without the balancer (Figure S1). This result is consistent with the idea that *eat-6(ad467)* is a reduction-of-function mutation [10] and suggests that a low dosage of the WT gene, in contrast to a high dosage introduced by an extrachromosomal array, can correct aldicarb sensitivity of the *ad467* mutant.

We also characterized pharyngeal electrical activity in animals with excess or reduced EAT-*6*. In contrast to the effect on aldicarb sensitivity, expression of *Ex[eat-6(+)]* in WT background did not change EPG (Figure 3b,f), which again points to distinctive functions of EAT-6 in the pharyngeal and locomotory circuits. Furthermore, *eat-6* RNAi altered EPG differently from that seen in *eat-6* mutants. In WT and mock-treated *rrf-3* control animals almost every excitatory postsynaptic potential (EPSP) triggered an action potential (AP) in the pharyngeal muscles (Figure 3b,c,h) [21]. Even the strong *eat-6(ad467)* mutation did not change the probability of AP generation, but reduced the rate of AP decay, which corresponds to a reduced amplitude of the recovery (R) peak in EPG (Figure 3b,d) [10]. In contrast, in *eat-6* RNAi worms about 50% of EPSPs failed to elicit APs in the pharyngeal muscles (Figure 3b,g). When APs were generated in *eat-6* RNAi worms, their R peaks were not reduced (Figure 3b,g). In fact, the mean relative amplitude of the R peak in the *eat-6* RNAi animals was even larger than that in WT and mock-treated control (Figure 3b). Outside of the pharynx, some effects of *eat-6* mutations and *eat-6* RNAi also differ. For example, the mutant worms showed no apparent motility defects [10], whereas RNAi animals were severely uncoordinated. These observations suggest that the phenotypes caused by the *ad467* mutation reflect allelic-specific defects. It is possible that the mutation alters specific enzyme properties, such as affinities for K^+^ and Na^+^ or voltage dependence, as shown for several mutations in mammalian homologs of *eat-6* [22], [23]. However, it remains possible that some differences between the phenotypes of *ad467* worms and that of RNAi-treated animals are due to resistance of some neurons to RNAi even in the *rrf-3* background.

### Cell-specific RNAi of *eat-6* in ACh neurons causes hypersensitivity to aldicarb

**Figure 4:**
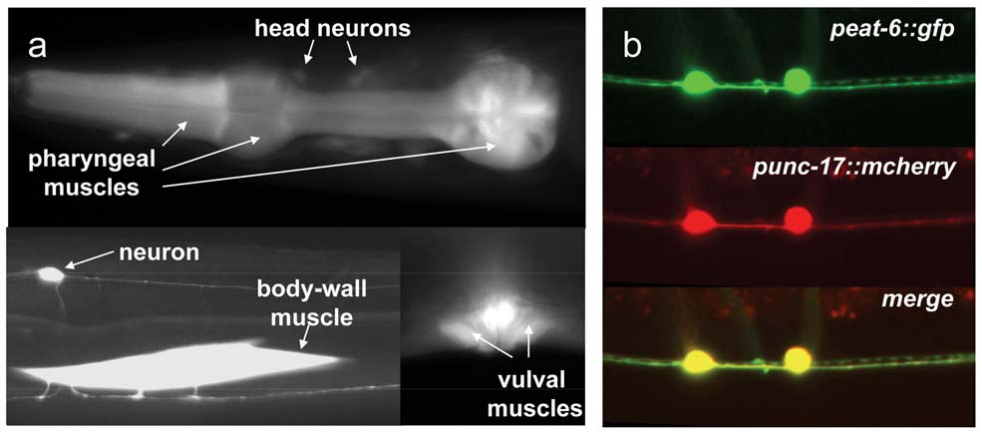
*eat-6* is expressed in muscles and neurons. (**a**) Photomicrographs of animals expressing GFP driven by the *eat-6* promoter. (**b**) Top, a photomicrograph of GFP driven by the *eat-6* promoter; middle, a photomicrograph of mCherry driven by the *unc-17* promoter; and bottom, merge of the top and middle images.

As the first step to determine cellular sites of EAT-6 function in ACh signaling, we constructed a GFP reporter under the control of the *eat-6* promoter (*peat-6::gfp*) and generated multiple transgenic lines from independent injections of this construct. Like that of many other GFP reporters, the expression of *peat-6::gfp* was mosaic, but was consistent in terms of cell types in at least three lines that were carefully examined in several generations. In agreement with a previous study [8], we observed GFP fluorescence in the pharyngeal, bodywall and vulval muscles. In addition, we found *eat-6* expression in many neurons, including the ventral cord ACh neurons, as well as in several head and tail neurons (Figure 4). This expression pattern raised the possibility that *eat-6* may regulate neurotransmission both pre- and postsynaptically.

**Figure 5:**
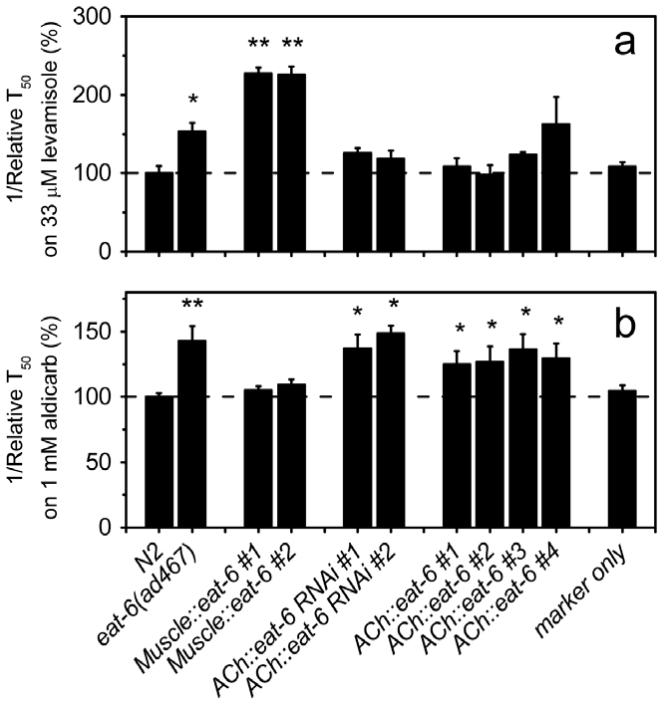
*eat-6* in both muscles and neurons regulates synaptic transmission. Synaptic transmission was assayed by scoring paralysis induced by levamisole (**a**) or aldicarb (**b**) at concentrations indicated in each panel. The relative reciprocal T_50_ values were calculated as described in Figure 2a. The error bars indicate SEM (n>3 replicates). * p<0.05, ** p<0.001, compared to WT. Student's *t*-test.

EAT-6 function at the postsynaptic site of ACh neurotransmission at the bodywall NMJs has been reported by others [8]. In agreement with the report [8], we observed that *eat-6(ad467)* mutant animals were hypersensitive to paralysis induced by levamisole, a specific agonist of the nicotinic ACh receptor UNC-29 (Figure 5a). Furthermore, like the *Ex[eat-6(+)]* transgene, a transgene expressing the WT *eat-6* cDNA under the bodywall muscle-specific *myo-3* promoter (*Muscle::eat-6*) also caused strong hypersensitivity to levamisole in the WT background (Figure 5). This result was unexpected, as Doi and Iwasaki reported successful rescue of the levamisole hypersensitivity of the *eat-6(ad467)* and *eat-6(ad601)* mutants by expressing *eat-6* in the muscles [8]. While the reason for the difference is not clear, one possible explanation could be dosage differences of *eat-6* in the transgenic arrays.

We went on to test whether *eat-6* also functions in the ACh neurons to regulate neurotransmission. We knocked down *eat-6* expression in the ACh neurons using cell-specific RNAi [24]. Both transgenic lines in which *eat-6* was silenced in the ACh neurons were hypersensitive to aldicarb, but not to levamisole (Figure 5a,b). These results suggest that, in addition to its role in the bodywall muscles, EAT-*6* in the ACh neurons may also influence ACh neurotransmission.

Next, we expressed the *eat-6* cDNA under the control of the promoter of the vesicular ACh transporter gene *unc-17* (*ACh::eat-6*). Like *Ex[eat-6(+)]* and *Muscle::eat-6, ACh::eat-6* did not correct aldicarb hypersensitivity or 5-HT resistance in *eat-6* mutant animals (Figure S2). However, four transgenic lines expressing *ACh::eat-6* in the WT background all showed strong hypersensitivity to aldicarb, but not to levamisole (Figure 5a,b). The *unc-17(e245)* mutant significantly reduces ACh release, and therefore is resistant to aldicarb-induced paralysis [25], [26]. When the *ACh::eat-6* transgene was crossed into the *unc-17(e245)* background, the transgenic animals became completely resistant to aldicarb (Figure S3). These results are consistent with the model that EAT-6 functions in both the bodywall muscles and the ACh neurons, and that either increased or decreased EAT-6 function may perturb ACh neurotransmission. However, since we were unable to rescue the aldicarb hypersensitivity of the *eat-6* mutants by expressing EAT-6 in the ACh neurons, other experiments are necessary to determine the mechanisms by which EAT-6 regulates presynaptic neurotransmission.

### The synaptic architecture of the *eat-6(ad467)* mutant

To probe for a possible role of *eat-6* in synaptic architecture we examined the expression and localization of GFP-tagged post- and presynaptic markers in *eat-6(ad467)* animals. We found that UNC-29::GFP fluorescence at the bodywall NMJs was slightly increased in the *ad467* animals relative to WT (Figure S4), although this difference was not statistically significant. This result is in agreement with a published study [8].

We also examined fluorescence of the GFP-tagged synaptic vesicle marker synaptobrevin *snb-1* expressed in a subset of the cholinergic motor neurons under the *unc-4* promoter [27]. No significant difference in SNB-1::GFP fluorescence was found between *ad467* and WT worms (data not shown), which is again consistent with the previous report [8]. These results indicate that the *eat-6(ad467)* mutation does not cause any gross defect in morphology of the NMJs.

**Figure 6:**
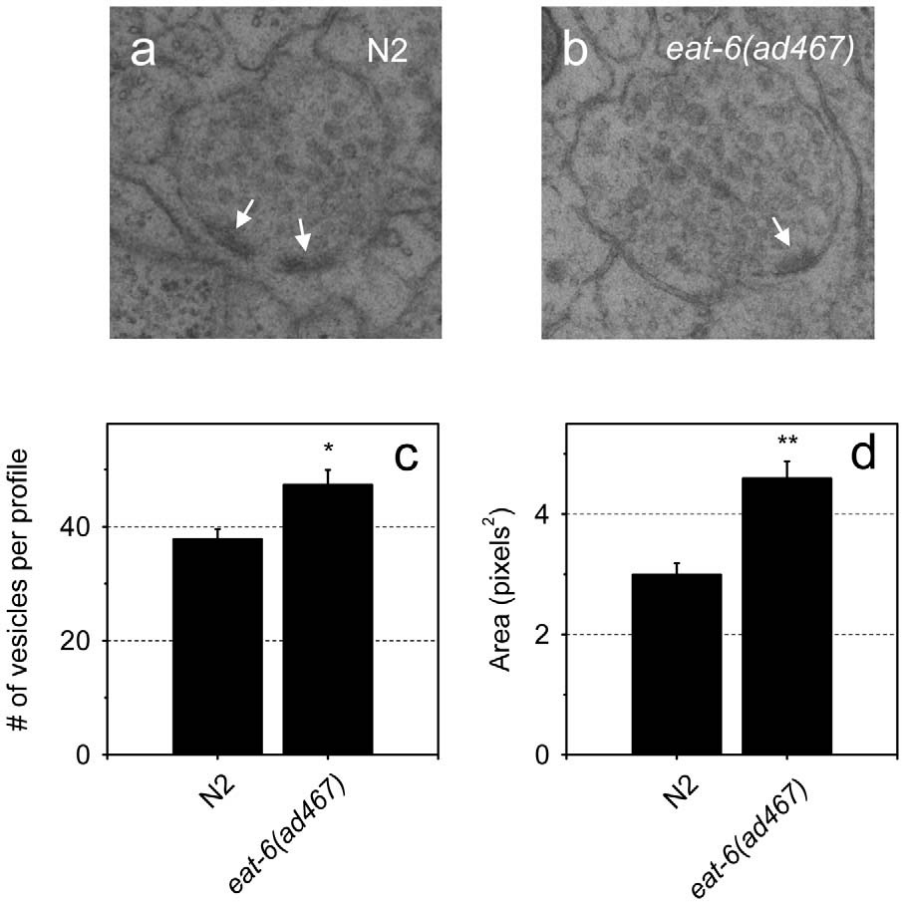
The *eat-6(ad467)* mutant accumulates more synaptic vesicles at ACh NMJs. (**a**) and (**b**) Representative synaptic profiles of WT and *ad467* mutant worms obtained by high-pressure freezing EM. Arrows point to the active zone. (**c**) The average number of synaptic vesicles per profile and SEM, * p<0.005. (**d**) The average area of the synaptic profile and SEM. ** p<0.001. The data were collected from 24 and 23 synaptic profiles, respectively, in WT and *ad467* worms. Four animals per each genotype were analyzed. Student's *t*-test.

To test whether hypersensitivity to aldicarb in the *eat-6(ad467)* mutant is resulted from alterations in synaptic vesicle formation or distribution, we carried out ultrastructural analysis of the ACh synapses by transmission electron microscopy (TEM). We found that the mean number of vesicles per synaptic profile was greater in the mutant relative to WT (Figure 6c). In *C. elegans*, the nerve processes in the nerve ring and along the ventral cord show large variations in axon diameter in correlation with vesicle content at synapses. The axon plasma membrane at a synaptic site is molded tightly around vesicle clusters, so the more vesicles, the larger the axon caliber [28], [29] (Hall, unpublished). Indeed, the mean area of the axon profile at ACh synapses in *eat-6* mutant animals is significantly larger than that in WT (Figure 6d). This result suggests that EAT-6 might play a role in regulation of the abundance of synaptic vesicles at the NMJs. The reason for the discrepancy between an increased number of synaptic vesicles revealed by EM analyses and the insignificant change in GFP-tagged synaptobrevin by fluorescence microscopy is not clear at present. One possibility could be that subtle changes in intensity of the GFP reporters may not be discernible with our experimental setting. Another attractive hypothesis would be less synaptobrevin per synaptic vesicle in *eat-6* mutants.

### EAT-6 acts upstream of EGL-30 Gαq in a genetic pathway

**Figure 7:**
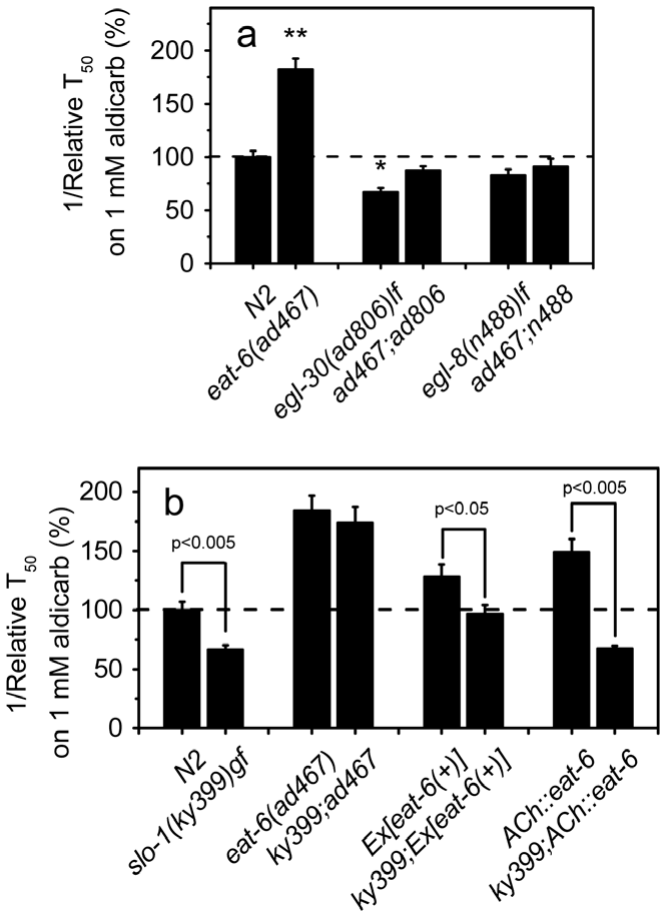
Genetic interaction between *eat-6* and *egl-30, egl-8* and *slo-1*. Relative reciprocal T_50_ values of paralysis induced by aldicarb. The error bars indicate SEM (n>3 replicates). * p<0.05, ** p<0.005, compared to WT. The p value between two tested groups is indicated on the top of the bars. In (**a**) the difference between *egl-30(ad806)* and *ad467;ad806* is significant (p<0.5). Student's *t* test.

To gain more insights into molecular mechanisms by which EAT-6 regulates synaptic transmission, we have sought for mutations in other genes that might block aldicarb hypersensitivity in the *eat-6(ad467)* mutant. Gαq EGL-30 promotes production of diacylglycerol (DAG), which facilitates ACh release from the ventral cord motor neurons [30], [31]. The *egl-30(ad806)* loss-of-function mutant animals are resistant to aldicarb [32]. We found that *egl-30(ad806)* eliminated aldicarb hypersensitivity of *ad467* (Figure 7a). The *egl-8* phospholipase Cβ is a downstream target of *egl-30* in the presynaptic pathway, and *egl-8* loss-of-function mutants show phenotypes similar, albeit weaker, to those of *egl-30* mutants [32], [33]. The *egl-8(n488)lf* mutation also suppressed hypersensitivity of the *eat-6* mutant to aldicarb (Figure 7a). These results suggest that *eat-6* acts upstream of *egl-30* in the genetic pathway. *egl-30* expression has been shown in neurons, pharyngeal and vulval muscles, but not in the bodywall muscles [34]. Interestingly, the *egl-30;eat-6* and *egl-8;eat-6* double mutants remained hypersensitive to levamisole (Figure S5).

There are no predicted voltage-gated Na^+^ channels in the *C. elegans* genome [35], and no Na^+^ APs are generated in *C. elegans* neurons [36]. Nevertheless, EAT-6 Na^+^,K^+^-ATPase may influence neurotransmitter release from the motor neurons by changing the resting membrane potential, and/or by influencing the activity of other ion channels or transporters. To explore a possible role of EAT-6 in this process, we analyzed the interaction between EAT-6 and SLO-1. SLO-1 is a large-conductance, Ca^2+^-activated K^+^ channel that acts presynaptically to repolarize active neurons, leading to closure of the voltage-gated Ca^2+^ channels and thus terminating neurotransmitter release [37]. Loss of *slo-1* function causes prolonged ACh release and hypersensitivity to aldicarb [37]. Conversely, worms carrying the *slo-1(ky399)* gain-of-function mutation are resistant to aldicarb [38], presumably because of a shortened duration of ACh synaptic release. When we crossed the *ad467* mutation into the *slo-1(ky399)* background, the double mutant remained as hypersensitive to aldicarb as the single *eat-6(ad467)* mutant (Figure 7b), in contrast to the *eat-6(ad467);egl-30(ad806)* double mutant that became resistant (Fig. 7a). This result suggests that EAT-6 activity is required for the gain-of-function effect of *slo-1(ky399)*.

Although overexpression of *eat-6* in the WT background caused hypersensitivity to aldicarb as did the *ad467* mutation (Figure 3a), the *slo-1(ky399)*;*Ex[eat-6(+)]* transgenic animals exhibited aldicarb sensitivity similar to that of WT animals (Figure 7b), suggesting that an excess of EAT-6 can compensate for or offset the effect of the *ky399* mutation. Similarly, the *ky399* mutation also suppressed aldicarb hypersensitivity induced by *ACh::eat-6* (Figure 7b). Therefore, although both reduction and excess of EAT-6 function cause hypersensitivity to aldicarb, their mechanisms differ.

### Exogenous 5-HT acts via *egl-30*, *tom-1* and *slo-1*

**Figure 8:**
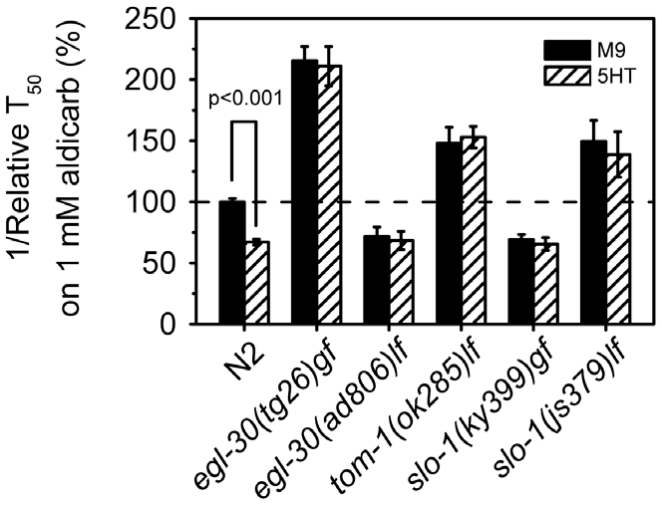
Mutants of *egl-30, tom-1 and slo-1* are insensitive to 5-HT treatment. Relative reciprocal T_50_ values and SEM of aldicarb-induced paralysis of worms pretreated with 5-HT (hatched bars) and that without 5-HT treatment. The values of 5-HT-treated WT worms and mutants are normalized to that of WT worms without 5-HT treatment. n>3 replicates. None of the tested mutants showed significant difference between 5-HT-treated and untreated worms, Student's *t* test.

Exogenous 5-HT inhibits the paralytic effects of aldicarb but does not affect the sensitivity to levamisole (data not shown) [17], suggesting that 5-HT regulates presynaptic ACh release. The resistance to 5-HT treatment observed in *ad467* and *eat-6* RNAi animals (Figures 2 and 3) could reflect a specific role of EAT-6 in 5-HT signaling or a general phenotype of aberrant synaptic transmission. To discriminate between these possibilities, we analyzed the effects of 5-HT on aldicarb-induced paralysis in mutants defective in different aspects of ACh neurotransmission. It has been reported that worms carrying *egl-30* loss- or gain-of-function mutations showed opposite egg-laying phenotypes, but were both resistant to stimulation of egg laying by 5-HT treatment [30]. Similarly, we found that although loss- and gain-of-function *egl-30* mutants were, respectively, resistant and hypersensitive to aldicarb [32], they were both insensitive to 5-HT treatment (Figure 8). Since aldicarb hypersensitivity of *eat-6(ad467)* worms was suppressed by *egl-30(ad806)*, but not by *slo-1(ky399)*, we tested whether 5-HT can modify aldicarb sensitivity in *slo-1* mutants. We found that both loss- and gain- of-function *slo-1* mutants were resistant to 5-HT treatment (Figure 8). Furthermore, mutants of the syntaxin-binding protein TOM-1, which regulates synaptic vesicle priming [39], were also resistant to 5-HT treatment (Figure 8). We conclude that mutations that disrupt regulated neurotransmitter release by either enhancing or attenuating it may compromise the effects of 5-HT treatment. Interestingly, WT animals expressing *Ex[eat-6(+)]* remained partially responsive to 5-HT treatment (Figure 3a), indicating that excessive amounts of normal EAT-6 protein does not disrupt 5-HT signaling. These results support the notion that 5-HT acts via presynaptic mechanisms to modulate synaptic transmission [17], and suggest that EAT-6 is a component in the pathway regulated by 5-HT.

### Deletion of the G protein-coupled 5-HT receptor SER-4 suppresses aldicarb hypersensitivity of *eat-6* mutants

**Figure 9:**
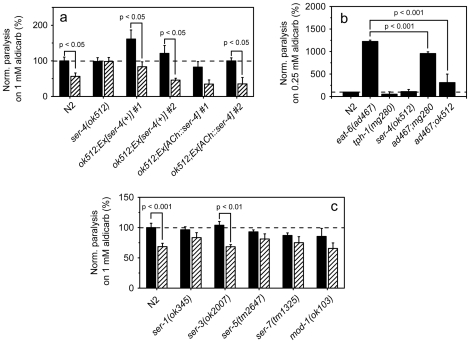
Role of 5-HT receptors in response to exogenous 5-HT. (**a**) The response to 5-HT treatment of *ser-4* mutants and the mutants expressing a WT *ser-4* transgene. Aldicarb-induced paralysis of worms pretreated with 5-HT (hatched bars) and those without 5-HT treatment (black bars) were scored by calculating the percentage of worms paralyzed after 60 min of exposure to aldicarb. The error bars indicate SEM (n>3 replicates). Noticeably, the aldicarb sensitivity of *ok512;Ex[ser-4(+)]* #1 worms without 5-HT treatment is significantly (p<0.05) higher than that of WT. (**b**) Mutations in *ser-4* and *tph-1* suppress aldicarb hypersensitivity of *eat-6* mutant worms. Aldicarb-induced paralysis was scored after 100 min of exposure to the drug. The error bars indicate SEM (n>3 replicates). (**c**) The response to 5-HT treatment of mutants of other 5-HT receptors. Aldicarb-induced paralysis of worms pretreated with 5-HT (hatched bars) and that without 5-HT treatment was scored by calculating the percentage of worms paralyzed after 60 min of exposure to the drug. The error bars indicate SEM (n>3 replicates). The values of 5-HT-treated WT worms and mutants are normalized to that of WT worms without 5-HT treatment. Student's t-test.

The *C. elegans* genome encodes one ionotropic 5-HT receptor and seven predicted G protein-coupled 5-HT receptors [40]. To identify receptors responsible for modulation of ACh neurotransmission by exogenous 5-HT, we analyzed available deletion mutants of these genes by the aldicarb assay. The sensitivity to aldicarb itself was not significantly changed in any of the tested 5-HT receptor mutants compared to that of WT (Figure 9a,c). However, the functional null allele *ser-4(ok512)* [41] was completely resistant to 5-HT treatment (Figure 9a). Transgenic expression of WT *ser-4* gene rescued 5-HT sensitivity of the *ser-4(ok512)* mutant (Figure 9a), indicating that SER-4 function is required for exogenous 5-HT to downregulate ACh neurotransmission. To determine whether SER-4 acts in the ACh neurons, we generated transgenic animals expressing *ser-4* cDNA specifically in these neurons (*ACh::ser-4*) in the *ok512* background. The 5-HT response in such animals was fully restored (Figure 9a). Deletion mutants of *ser-1*, *ser-5*, *ser-7* and *mod-1* also showed reduced sensitivity to 5-HT treatment (Figure 9c), suggesting that multiple 5-HT receptors are at work at the NMJs.

To elucidate the relationship between 5-HT signaling and EAT-6, we generated an *eat-6(ad467);ser-4(ok512)* double mutant. Surprisingly, although the *ser-4(ok512)* mutant itself showed WT sensitivity to aldicarb, the *ser-4* mutation significantly suppressed aldicarb hypersensitivity of the *eat-6(ad467)* mutant (Figure 9b; Figure S6). Likewise, null mutants of the tryptophan hydroxylase gene *tph-1* that cannot biosynthesize serotonin [42] showed WT sensitivity to aldicarb, but the *tph-1* mutation significantly attenuated aldicarb hypersensitivity of the *eat-6(ad467)* mutant (Figure 9b). These results indicate that there is indeed a genetic interaction between *eat-6* and endogenous 5-HT signaling.

### Specific serotonergic neurons are responsible for stimulatory and inhibitory 5-HT inputs

Suppression of aldicarb hypersensitivity of the *eat-6(ad467)* mutant by the *ser-4* mutation raises a possibility for a stimulatory 5-HT input to ACh neurotransmission. Genetic analysis of *C. elegans* has demonstrated that distinct serotonergic neurons regulate specific aspects of behavior [43] and that multiple 5-HT receptors may exert opposite effects on the same behavior [44]. To understand how serotonergic neurons regulate ACh neurotransmission, we analyzed the role of 5-HT signal from specific neurons. In *C. elegans* hermaphrodites, 5-HT is primarily produced in the ADF chemosensory neurons, the NSM secretory neurons in the head region, and the HSN motor neurons responsible for egg laying [42], [45]. In addition, the single RIH neuron and a pair of the AIM interneurons can absorb extracellular 5-HT via the serotonin reuptake transporter (SERT) MOD-5, but cannot synthesize it (Kullyev et al., unpublished).

**Figure 10:**
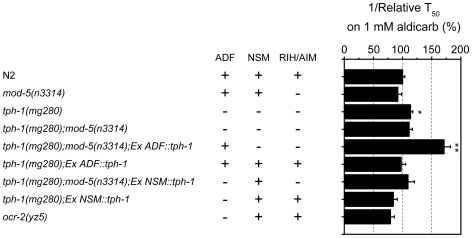
5-HT from the ADF and the NSM/RIH/AIM neurons antagonistically regulates ACh neurotransmission. Relative reciprocal T_50_ values of aldicarb-induced paralysis of worms expressing the *tph-1* gene in specific neurons. The error bars indicate SEM (n>3 replicates). * p<0.05, ** p<0.005, compared to WT by Student's *t*-test. The presence or absence of 5-HT in specific neurons is designated by “+” or “-”, respectively.

To test for contributions of specific serotonergic neurons in modulation of neurotransmission, we expressed *tph-1* cDNA either in the ADF neurons or the NSM neurons in a *tph-1* null background. To prevent neurons from absorbing extracellular 5-HT, we introduced individual neuron-specific *tph-1* transgenes into the *tph-1;mod-5* double mutant. We confirmed the presence of 5-HT in specific neurons by staining the transgenic animals with anti-5-HT antibodies (data not shown). When the *tph-1* transgene was expressed in the ADF neurons in the *tph-1;mod-5* background, the worms became strongly hypersensitive to aldicarb (Figure 10). Conversely, when 5-HT was excluded from the ADF neurons, but present in the other neuronal classes, the worms were slightly resistant to aldicarb, as compared to WT (Figure 10). A similar degree of resistance was also observed in the TRPV channel *ocr-2* mutants, which has reduced 5-HT specifically in the ADF neurons [46]. These results indicate that endogenous 5-HT released from the ADF neurons stimulates ACh synaptic transmission at the NMJs, whereas 5-HT from the NSM/RIH/AIM neurons inhibits the stimulatory 5-HT inputs.

## Discussion

FHM was the first human disease linked to mutations in a specific subunit of Na^+^,K^+^-ATPase [47]. Later it became evident that the same α2 subunit is associated with a much broader spectrum of pathologies, including other forms of migraine, epilepsy and ataxia [48]. In addition, mutations in the α3 subunit have been found in patients suffering from rapid-onset dystonia Parkinsonism [49]. Finally, genetic variations in all three subunits of Na^+^,K^+^-ATPase expressed in brain have been linked to bipolar disorders [50]. Cellular and molecular mechanisms by which specific mutations cause disease phenotypes remain poorly understood. In this study, we showed that EAT-6, a *C. elegans* homolog of human FHM2, controls excitatory synaptic transmission and its modulation by 5-HT, both of which are altered in migraine patients.

### Both reduction and excess of EAT-6 Na^+^,K^+^-ATPase alter synaptic transmission

There is an ongoing debate in the literature on whether FHM2 mutations cause pathology by a loss- or a gain-of function mechanism [51]. Several FHM2 alleles have been studied by expressing the mutant genes in oocytes or cell culture. Some of the FHM2 mutations clearly result in reduced function of the enzyme, ranging from a complete loss of pump current to subtle changes in the voltage gating, cation affinities or enzyme turnover [52]. On the other hand, certain effects of FHM2 mutations argue for gain-of-function activity [51]. In this study, we found that both silencing *eat-6* by RNAi and *eat-6* overexpression increased sensitivity to an ACh esterase inhibitor, suggesting enhanced ACh neurotransmission. These findings may provide a clue to reconcile some paradoxical observations of the pathogenic FHM2 alleles.

A primary cause of the migraine aura is “cortical spreading depression” (CSD), a wave of transient neuronal depolarization that spreads over the brain cortex and presumably activates the trigeminovascular system resulting in migraine headache. Mutations in all FHM genes have been predicted to cause increased levels of the neurotransmitter glutamate and K^+^ in the synaptic cleft leading to increased propensity for CSD [53]. In the case of FHM1 this hypothesis has been validated by analysis of knock-in mice [54]. However, for FHM2 such studies have not yet been reported. Our genetic analyses of EAT-6 supports a model that the FHM2 Na^+^,K^+^-ATPase may regulate synaptic transmission both pre- and postsynaptically.

What are the molecular mechanisms by which *eat-6* mutations may enhance ACh neurotransmission at the NMJ? A postsynaptic role for *eat-6* has been shown convincingly before by others [8]. Our ACh neuron-specific *eat-6* RNAi results suggest that *eat-6* may also function in the ACh neurons to regulate neurotransmission. We observed an increased number of synaptic vesicles in the *eat-6(ad467)* mutant. Although the mechanism by which Na^+^,K^+^-ATPase regulates the abundance of synaptic vesicles is unclear, our genetic analyses shed some light on a possible role of EAT-6 in regulation of ACh neurotransmission. The aldicarb hypersensitivity by reduced EAT-6 function could be, at least in part, ascribed to exaggeration of EGL-30 Gαq signaling to the EGL-8 phospholipase, because a reduction of *egl-30* and *egl-8* function suppressed aldicarb hypersensitivity of the *eat-6(ad467)* mutant. Moreover, we found that a *slo-1(gf)* mutant overexpressing EAT-6 exhibited WT aldicarb sensitivity, although *slo-1(gf);eat-6* double mutant worms were as hypersensitive to aldicarb as the *eat-6* single mutant. These results suggest that, while both reduced and excessive EAT-6 function may increase ACh neurotransmission, their mechanisms differ. One possibility would be that the hyperactivity of SLO-1 compensates for the effects caused by increased EAT-6 function, whereas a reduced transmembrane K^+^ gradient in *eat-6* mutant animals renders this hyperactive SLO-1 channel inefficient. It has been reported that EAT-6 possesses a novel function that is unrelated to the pump function of this enzyme [8]. We speculate that overexpression and the mutation of EAT-6 differentially affect this yet undefined EAT-6 function, which in turn perturb particular aspects of cellular mechanisms in ACh neurotransmission. However, since we were unable to rescue the aldicarb hypersensitivity of the *eat-6* mutants by expressing EAT-6 in the ACh neurons, other experiments are necessary to determine its role in regulation of presynaptic neurotransmission.

### Opposing 5-HT inputs to ACh neurotransmission

We demonstrated that *eat-6* not only regulates ACh neurotransmission, but it is also involved in modulation of this process by exogenous and endogenous 5-HT. Our observation of *eat-6* mutants as well as mutants of many synaptic components being completely resistant to 5-HT treatment suggests that *eat-6* is one component mediating the inhibitory input of 5-HT to ACh neurotransmission. This result could be relevant to the therapeutic effects of drugs targeting 5-HT receptor subtypes in the treatment of migraine [55].

Analysis of the G protein-coupled 5-HT receptor *ser-4* revealed dual stimulatory and inhibitory 5-HT inputs to ACh neurotransmission. One the one hand, *ser-4* appears to be the primary receptor that mediates inhibition of ACh neurotransmission by exogenous 5-HT. On the other hand, deletion of *ser-4* significantly suppressed aldicarb hypersensitivity of the *eat-6(ad467)* mutant, suggesting that SER-4 activity is required for the increased aldicarb sensitivity of the *eat-6* mutant. Such dual activities of 5-HT receptors have been observed in mammals. For example, the 5-HT1A receptor can both stimulate and inhibit adenylyl cyclase [56]. Further experiments are required to discern the precise mechanisms of SER-4 action in stimulatory and inhibitory 5-HT signaling. One thought is that *ser-4* may function in different neurons to inhibit or stimulate ACh neurotransmission. In rodents, *in vivo* binding assays showed that the 5-HT1A receptor expressed in different neurons in the CNS has different affinities to 5-HT. It is plausible that different levels of 5-HT differentially activate SER-4 in different neurons to modulate ACh neurotransmission. Consequently, the *ser-4* mutation would abolish both the inhibitory and stimulatory 5-HT inputs to ACh neurotransmission, while loss of EAT-6 may selectively block the inhibitory 5-HT input. Interestingly, we found that specific serotonergic neurons are responsible for stimulatory and inhibitory 5-HT inputs to ACh neurotransmission at the bodywall NMJs. Genetic analyses in *C. elegans* have demonstrated that 5-HT released from specific neurons regulates specific behavior [43], while many 5-HT receptors synergistically or antagonistically regulate the same behavior [41], [44]. It may be sensible that different serotonergic neurons regulate different receptor subtypes at different cellular targets coordinating synaptic activity according to dynamic changes in the environment.

## Materials and Methods

### Strains

*C. elegans* strains were maintained at 20°C as described [57]. Wild-type (WT) animals were the Bristol Strain *N2*. Mutants used were: AQ279 *unc-29(x29);Ex[unc-29::gfp;rol-6(d)]*, CX6741 *tph-1(mg280);Ex[ADF::tph-1]*, CX7749 *tph-1(mg280);Ex[NSM::tph-1]*, DA1239 *eat-6(ad467)*;*adEx1239*[*eat-6(+); rol-6(d)*], *eat-6(ad467)*, *eat-6(ad601)*, *eat-6(ad792)*, *eat-6(ad997)*, VC836 *eat-6(ok1334)/nT1[qIs51]*, *egl-8(n488)*, *egl-30(ad806)*, *egl-30(tg26), mod-1(ok103), mod-5(n3314)*, MT15434 *tph-1(mg280)*, NM670 *Ex*[*punc-4::snb-1::gfp;lin-15(+)*], *ocr-2(yz5)*, *rrf-3(pk1426), ser-1(ok345), ser-3(ok1995), ser-3 (ok2007), ser-4(ok512), ser-5(tm2647), ser-5(tm2654), ser-7(tm1325), slo-1(js379), slo-1(ky399), tom-1(ok285), unc-17(e245).*

The following *eat-6(ad467)* double mutants were generated in this study: *eat-6(ad467);egl-30(ad806), eat-6(ad467);egl-8(n488), eat-6(ad467);slo-1(ky399), eat-6(ad467);tph-1(mg280), eat-6(ad467);ser-4(ok512), eat-6(ad467);nT1.* In the case of the deletion alleles *n488*, *mg280* and *ok512* that can be identified by PCR, we first isolated homozygous *ad467* based on its slow pharyngeal pumping, small body size and fewer eggs (all these phenotypes manifest in *eat-6* RNAi worms), then isolated the homozygous deletion mutations. In the case of the missense alleles *ky399* and *ad806*, we first isolated cross-progeny showing the phenotypes of the homozygous *ky399* (uncoordinated locomotion [38]), and *egl-30* (sluggish movement and an increased number of eggs in the uterus [30]), and then picked homozygous *ad467* animals from their progeny. To construct the *ad467;nT1* strain, we generated males in the *ok1334;nT1* strain, which carries the homozygous *ok1334* mutation maintained by a single copy of the *nT1* translocation marked by GFP. These males were crossed with *ad467* hermaphrodites. The isolation of the homozygous *ad467* progeny carrying *nT1* was based on two criteria: first, worms should not carry the *ok1334* deletion as determined by PCR; second, as the strain carries a single copy of *nT1*, the progeny that do not carry *nT1* should show the *ad467* phenotypes. All the missense alleles were confirmed by sequencing.

### RNAi experiments

The *rrf-3* strain with enhanced neuronal sensitivity to RNA interference [58] was used to silence *eat-6*, as described [59]. Briefly, NGM plates containing 6 mM IPTG were seeded with bacterial cultures expressing the empty vector L4440 or *eat-6* RNAi construct from the RNAi feeding library constructed by J. Ahringer's group at the University of Cambridge and incubated overnight. 5–7 gravid *rrf-3* hermaphrodites were allowed to lay eggs on these plates for one day and removed afterwards. The progeny on the plates were analyzed when they became adults after three days.

### Constructs and transgenes

All the constructs were generated by PCR, and purified PCR fragments were used to generate transgenic animals. Unless specified otherwise, *pinx-16::gfp*, which is expressed in the intestine [60], was used as a transformation marker. The transcriptional reporter *peat-6::gfp* was generated by fusion of 1548 bp upstream of the translation start of *eat-6*, the same promoter element contained in *Ex[eat-6(+)]* [10], with the sequence of GFP and UNC-54 3′-untranslated regulatory element (UTR) in the plasmid pPD95.75 (from A. Fire, Stanford University School of Medicine, Stanford, CA). For tissue-specific expression full-length *eat-6* cDNA was amplified from RNA isolated from WT animals by RT-PCR (SuperScript III First-Strand Synthesis System, Invitrogen) and fused with *unc-54* 3′ UTR and either the *unc-17* [25] or *myo-3* [61] promoter. *punc-17::mCherry* was generated by fusion of the *unc-17* promoter element with the sequence of mCherry and *unc-54* 3′-UTR. The *Ex[ser-4(+)]* transgene has been described previously [41]. For cell-specific expression in ACh neurons full-length *ser-4* cDNA amplified from WT RNA was fused with the *unc-17* promoter and *unc-54* 3′ UTR. The construction and characterization of the transgenes expressing *tph-1* in the ADF (under the *srh-142* promoter) or NSM (under the *ceh-2* promoter) neurons have been described [43].

To knock down *eat-6* expression specifically in ACh neurons, we used a cell-specific RNAi approach [24]. Briefly, an *eat-6* exon-rich 1089 bp genomic fragment, which was used to generate the *eat-6* RNAi clone in the RNAi feeding library [59], was fused in the forward and reverse orientations by two different PCR reactions with the *unc-17* promoter and *unc-54* 3′ UTR, so that the *eat-6* gene fragment can be transcribed by the *unc-17* promoter in the sense and antisense orientations. The two constructs were coinjected into worms in equal concentrations (∼100 ng/µl).

### Fluorescence microscopy

GFP fluorescence was monitored using a 63× objective lens with an AxioImager Z1 miscroscope (Zeiss). Images were captured at 2×2 binning with an AxioCam MR digital camera using the AxioVision software (Zeiss). The neutral density filter #5 of the microscope was used to reduce the intensity of fluorescence excitation light to mitigate bleaching. The same exposure time (500 ms) was used for all samples. The ImageJ software (NIH) was used for image analysis.

### Drug-induced paralysis assays

Sensitivity to drugs was determined by scoring paralysis after placing the animals on plates containing aldicarb or levamisole (Sigma) at the concentrations indicated in the figures. L4 stage animals were transferred to plates seeded with food and incubated at 20°C overnight, and resultant young adults were analyzed. An animal was scored as being paralyzed if no movement was detected after prodding with a platinum wire. In all experiments except that in Figure 9a,c, the time course of paralysis was measured by scoring percentage of animals paralyzed on the plates every 20 minutes over a three-hour period. To assay the inhibition of aldicarb effects by serotonin, animals were first incubated for 2 h on plates containing 5 mg/ml of serotonin creatine sulfate (Sigma), and were then transferred to plates containing both aldicarb (1 mM) and serotonin (5 mg/ml), and the time course of paralysis was assayed. For each genotype at least 3 independent trials were carried out, each performed in duplicate with 10 animals per assay plate. The results of mutants were compared with that of WT animals assayed in parallel.

### Egg laying assay

Egg-laying behavior was evaluated by two well-established methods (18, 41). In both cases L4 stage animals were picked onto fresh plates seeded with food and allowed to develop at 20°C, and one-day old adults were analyzed. To test the response to 5-HT, animals were placed individually into wells of a 96-well plate, with each well containing 100 µl of a solution (M9 buffer or M9 buffer containing 5 mg/ml of serotonin). The number of eggs laid was scored after 1 h. To score the number of eggs carried in the uterus, individual one-day old adults were placed into a drop of solution containing commercial bleach and 2N NaOH on a glass slide. The bleach solution dissolved the body of the adult animal, and eggs, which were protected by their eggshells, were scored immediately. Eight animals per genotype for each condition were assayed in each trial.

### Extracellular EPG recording

Extracellular recording of the electrical activity of the pharynx was made from intact worms as previously described [62]. One-day old adult worms were incubated in M9 buffer containing 10 mM 5-HT to stimulate pharyngeal pumping. The signals were acquired with an Axopatch 200B amplifier (Molecular Devices, Sunnyvale, CA) in voltage-clamp mode at 10 kHz sampling rate and were digitized using a Digidata 1322A AD/DA acquisition system controlled by a PC running Clampex 10.0 software (Molecular Devices), and analyzed using pClamp 10.0 software. The EPG reflects the time derivative of the membrane potential of the pharyngeal muscles. The EPG of WT worms consists of a large positive E peak, corresponding to muscle excitation, a plateau phase, and a large negative R spike, corresponding to muscle repolarization. In addition, recording EPG allows us to monitor neuronal inputs to the muscles. In particular, a small positive peak preceding the E peak represents an excitatory post-synaptic potential (EPSP) [21]. It results from activation of nicotinic receptors in the muscle membrane by ACh released from the MC neuron [63]. The amplitudes of the E and R spikes were measured in each of the first 5 action potentials recorded from each of 15–20 worms per genotype. Data collected from each individual worm were pooled together, and the mean R/E ratio was calculated for each genotype. To determine the percentage of the failed EPSPs, recording has been done till at least 30 action potentials were generated. All but the last EPSP in the series were counted, and those that were not immediately followed by the E spike were considered failed.

### EM analysis

For transmission EM one-day old adult animals were fixed by high-pressure freezing and freeze-substitution, using 2% osmium tetroxide in acetone as the primary fixative [64]. Serial sections (80 nm thickness) of fixed animals were collected on copper slot grids and stained with 4% uranyl acetate in 70% methanol, followed by washing and incubating with lead citrate. Images were captured using a Philips CM10 transmission electron microscope at 80 kV equipped with a Morada 11 MPixel TEM CCD camera driven by the iTEM software (Olympus Soft Imaging Solutions). A set of serial sections containing a presynaptic density was defined as a synapse, and the number of vesicles per synaptic profile was calculated in the middle section of each set using NIH ImageJ software. Neuronal classes (VA or VB, both cholinergic; or VD, GABAergic) were identified by tracing the processes along the anterior ventral cord in a series of about 500 serial sections per animal [65].

### Statistical analyses

Statistical analyses were performed using Origin 7.0 software (OriginLab Corporation, Northampton, MA). Significance was tested using an unpaired two sample Student's *t* test. Throughout the article data are expressed as mean ± SEM.

## Supporting Information

Figure S1 A single copy of the genomic *eat-6* gene rescues the aldicarb hypersensitivity of the *eat-6(ad467)* mutant. Relative reciprocal T_50_ values of aldicarb-induced paralysis of worms with different ratios of WT to mutant *eat-6* gene. An extra copy of the *eat-6*(+) locus was introduced by the *nT1* translocation into the *eat-6 (ok1334)* deletion background or into the *eat-6(ad467)* background. Heterozygous *ad467* animals were included as a control. The genotypes of the tested strains are schematically represented at left. The chromosomes IV and V are shown as long black and white bars, respectively. A short black bar represents the WT *eat-6* gene, the horizontally hatched short bar: the *eat-6(ad467)* allele, and the white short bar: the *eat-6(ok1334)* allele. The short obliquely hatched bar represents the *gfp* marker. The error bars indicate SEM (n>3 replicates).
(0.48 MB TIF)

Figure S2 Expression of *eat-6* cDNA in ACh neurons or body-wall muscles does not rescue the aldicarb hypersensitivity of *eat-6* mutants. Relative reciprocal T_50_ values of aldicarb-induced paralysis of worms pretreated with 5-HT (hatched bars) and those without 5-HT treatment (black bars). The values of 5-HT-treated WT worms and mutants are normalized to that of WT worms without 5-HT treatment. The error bars indicate SEM (n>3 replicates).
(0.21 MB TIF)

Figure S3 Time courses of aldicarb-induced paralysis of the *unc-17(e245)* and *unc-17(e245)*;*ACh::eat-6* transgenic animals. The error bars indicate SEM (n = 3 replicates).
(0.15 MB TIF)

Figure S4 Intensity of UNC-29::GFP fluorescence in *eat-6(ad467)* background, as compared to WT. Measurements were performed in 18 *ad467* and 47 WT worms. In each worm, an anterior section of the ventral cord (∼100 µm length) was photographed. The values of punctal fluorescence intensity were calculated from profiles drawn across the middle of the puncta along the ventral cord of individual worms. The error bars indicate SEM between worms.
(0.36 MB TIF)

Figure S5 Mutations in *egl-30* or *egl-8* do not suppress hypersensitivity of the *eat-6(ad467)* worms to levamisole. Relative reciprocal T_50_ values of paralysis were calculated as shown in Fig. 2. The error bars indicate SEM (n>3 replicates). * p<0.05, ** p<0.005, compared to WT.
(0.41 MB TIF)

Figure S6 Time courses of aldicarb-induced paralysis of *eat-6* and *eat-6;ser-4* mutants on1 mM (a) and 0.25 (b) mM aldicarb. The difference between the two hypersensitive strains, which is not significant on 1 mM of the drug, is clearly resolved on the lower concentration. The error bars indicate SEM (n>3 replicates).
(0.30 MB TIF)
