# Supplementary figures and images for: A Homolog of FHM2 Is Involved in Modulation of Excitatory Neurotransmission by Serotonin in C. elegans
Source: PLoS One. 2010 Apr 28;5(4):e10368. doi: 10.1371/journal.pone.0010368 (PMC2860991; doi:10.1371/journal.pone.0010368)

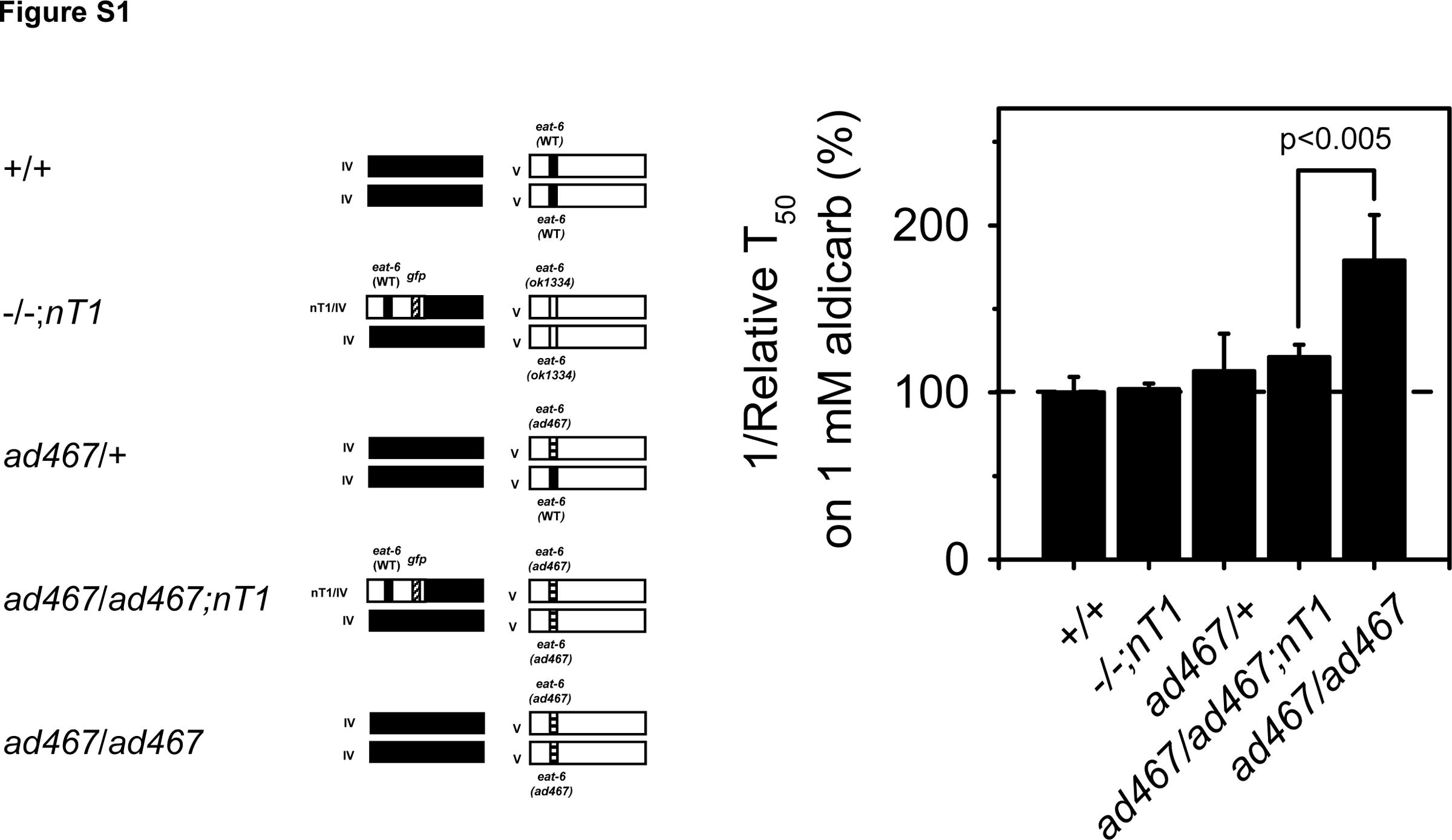

Supplement: Figure S1 — A single copy of the genomic eat-6 gene rescues the aldicarb hypersensitivity of the eat-6(ad467) mutant. Relative reciprocal T50 values of aldicarb-induced paralysis of worms with different ratios of WT to mutant eat-6 gene. An extra copy of the eat-6(+) locus was introduced by the nT1 translocation into the eat-6 (ok1334) deletion background or into the eat-6(ad467) background. Heterozygous ad467 animals were included as a control. The genotypes of the tested strains are schematically represented at left. The chromosomes IV and V are shown as long black and white bars, respectively. A short black bar represents the WT eat-6 gene, the horizontally hatched short bar: the eat-6(ad467) allele, and the white short bar: the eat-6(ok1334) allele. The short obliquely hatched bar represents the gfp marker. The error bars indicate SEM (n>3 replicates). (0.48 MB TIF) [file pone.0010368.s001.tif]

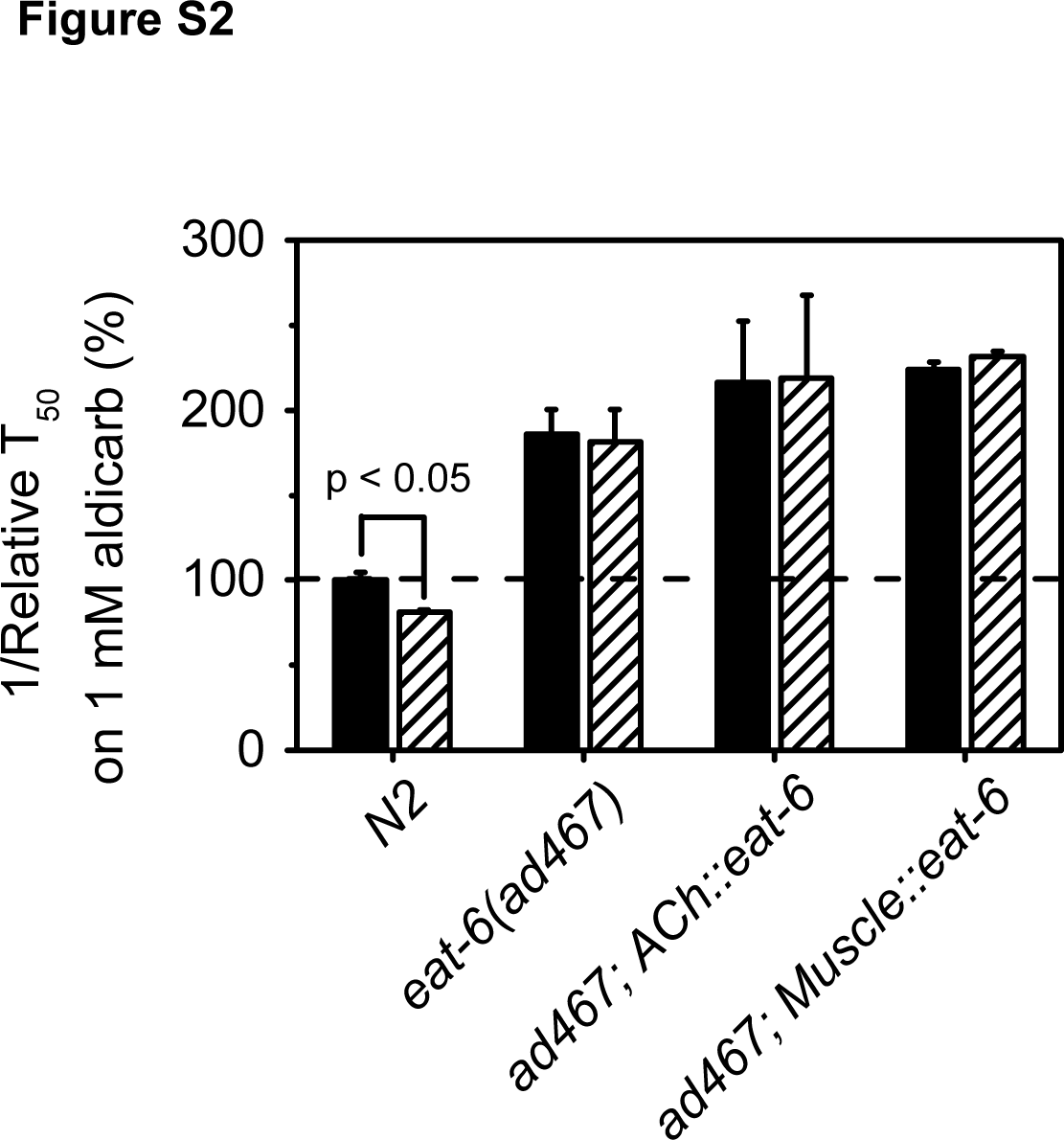

Supplement: Figure S2 — Expression of eat-6 cDNA in ACh neurons or body-wall muscles does not rescue the aldicarb hypersensitivity of eat-6 mutants. Relative reciprocal T50 values of aldicarb-induced paralysis of worms pretreated with 5-HT (hatched bars) and those without 5-HT treatment (black bars). The values of 5-HT-treated WT worms and mutants are normalized to that of WT worms without 5-HT treatment. The error bars indicate SEM (n>3 replicates). (0.21 MB TIF) [file pone.0010368.s002.tif]

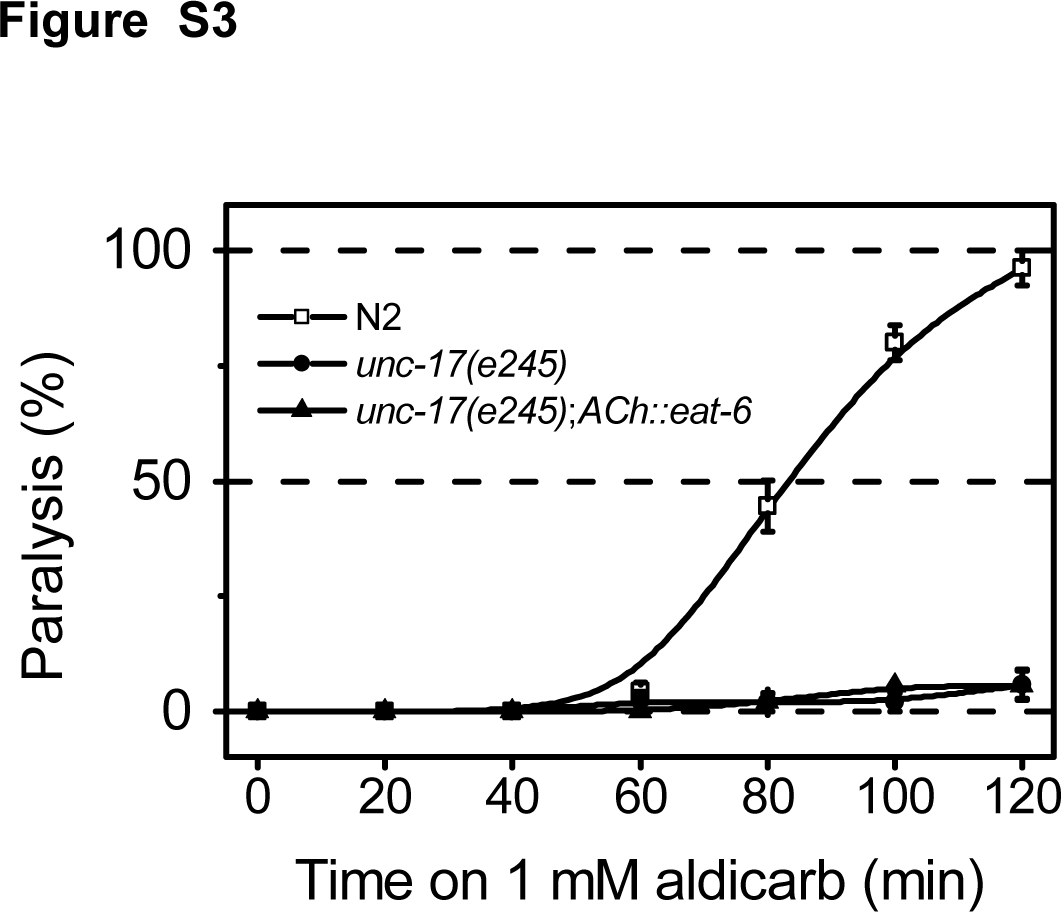

Supplement: Figure S3 — Time courses of aldicarb-induced paralysis of the unc-17(e245) and unc-17(e245);ACh::eat-6 transgenic animals. The error bars indicate SEM (n = 3 replicates). (0.15 MB TIF) [file pone.0010368.s003.tif]

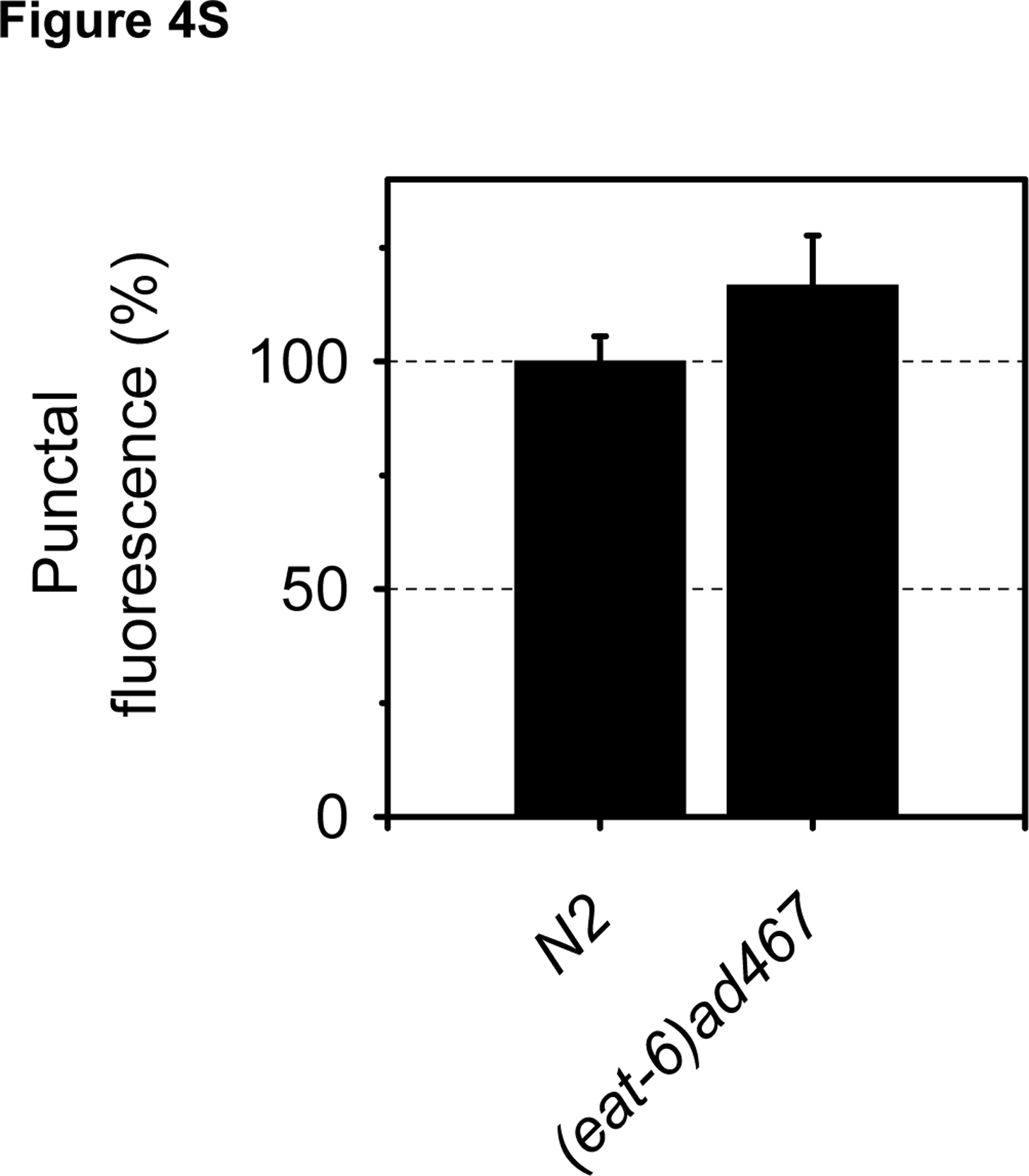

Supplement: Figure S4 — Intensity of UNC-29::GFP fluorescence in eat-6(ad467) background, as compared to WT. Measurements were performed in 18 ad467 and 47 WT worms. In each worm, an anterior section of the ventral cord (∼100 µm length) was photographed. The values of punctal fluorescence intensity were calculated from profiles drawn across the middle of the puncta along the ventral cord of individual worms. The error bars indicate SEM between worms. (0.36 MB TIF) [file pone.0010368.s004.tif]

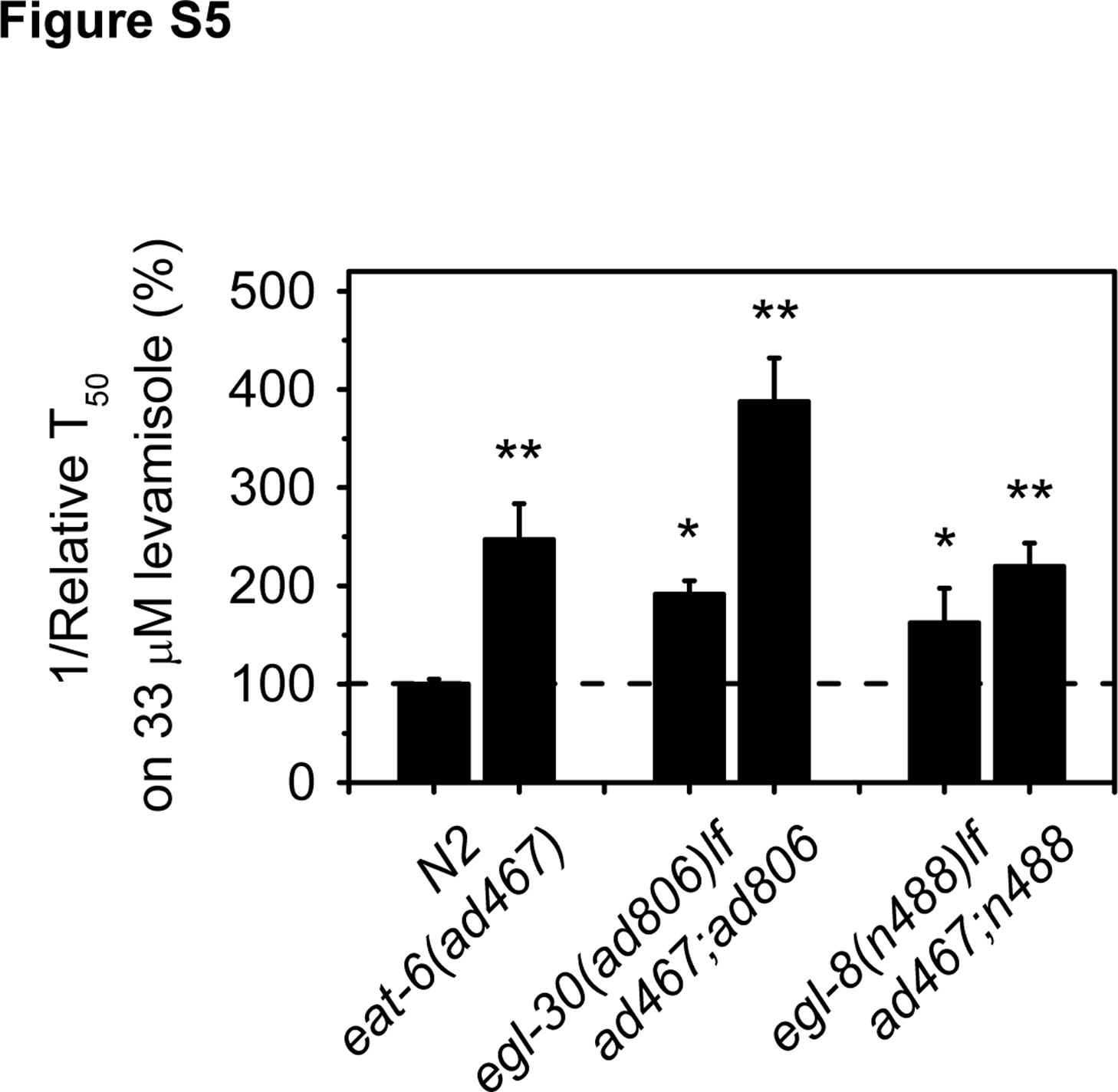

Supplement: Figure S5 — Mutations in egl-30 or egl-8 do not suppress hypersensitivity of the eat-6(ad467) worms to levamisole. Relative reciprocal T50 values of paralysis were calculated as shown in Fig. 2. The error bars indicate SEM (n>3 replicates). * p<0.05, ** p<0.005, compared to WT. (0.41 MB TIF) [file pone.0010368.s005.tif]

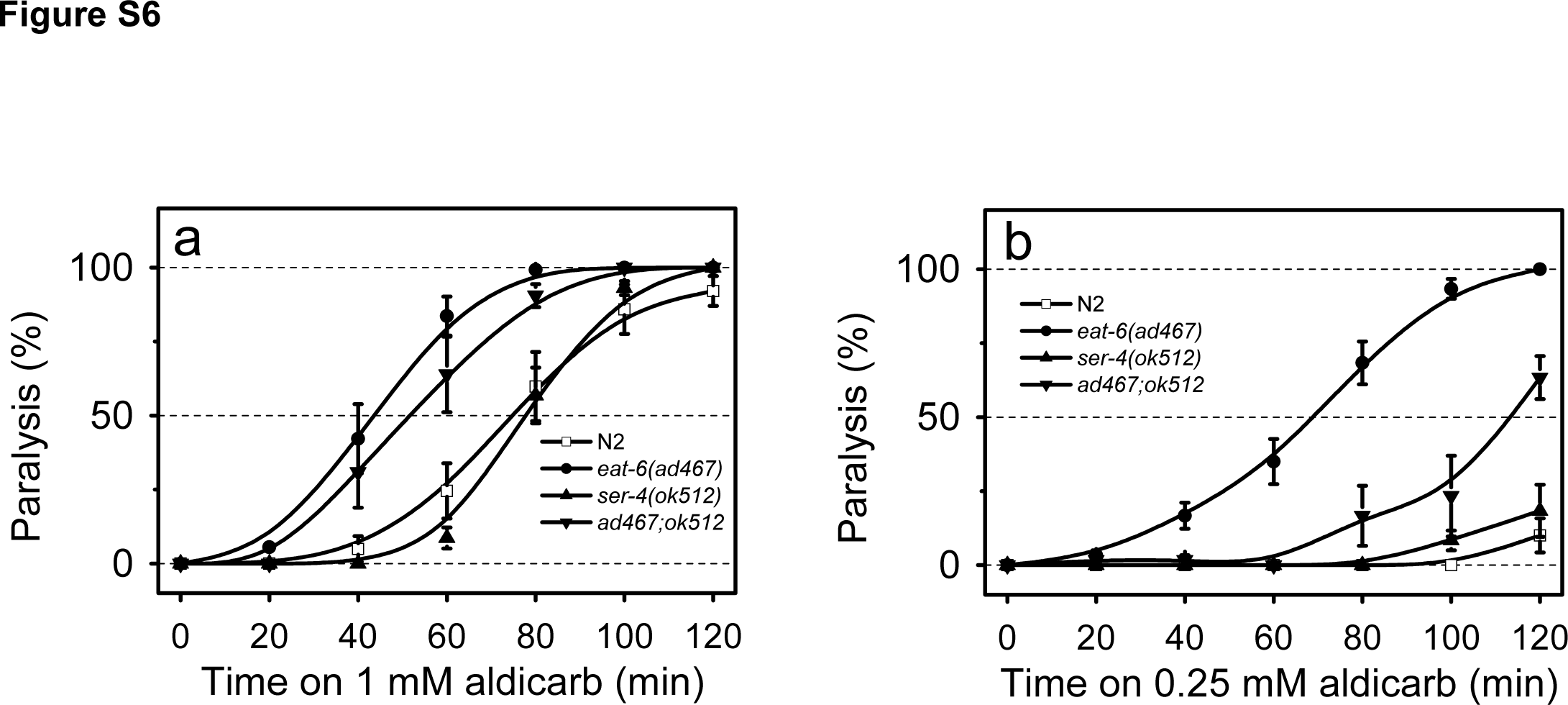

Supplement: Figure S6 — Time courses of aldicarb-induced paralysis of eat-6 and eat-6;ser-4 mutants on1 mM (a) and 0.25 (b) mM aldicarb. The difference between the two hypersensitive strains, which is not significant on 1 mM of the drug, is clearly resolved on the lower concentration. The error bars indicate SEM (n>3 replicates). (0.30 MB TIF) [file pone.0010368.s006.tif]
